# Oncological outcomes post focal low‐dose‐rate brachytherapy in low‐intermediate risk prostate cancer

**DOI:** 10.1002/bco2.70129

**Published:** 2026-02-11

**Authors:** Mohammadmehdi Adhami, Jeremy Cheng, Elliot Anderson, Lloyd Smyth, Cate Davey, Thang Nguyen, Richard O'Sullivan, Andrew Ryan, Nathan Lawrentschuk, Jeremy Grummet, Andrew See

**Affiliations:** ^1^ Department of Urology The Alfred Hospital Melbourne Victoria Australia; ^2^ School of Translational Medicine Monash University Melbourne Victoria Australia; ^3^ Icon Cancer Centre Melbourne Victoria Australia; ^4^ Department of Urology The Royal Melbourne Hospital Melbourne Victoria Australia

**Keywords:** focal brachytherapy, focal therapy, multiparametric MRI, prostate cancer, PSA velocity, transperineal prostate biopsy

## Abstract

**Objectives:**

To prospectively evaluate oncological control, pathological progression, and its predictors following focal low‐dose‐rate (LDR) brachytherapy for low‐intermediate risk prostate cancer (PCa).

**Patients and methods:**

LIBERATE is a prospective, multi‐centre clinical registry of patients who have undergone focal LDR brachytherapy for low‐intermediate risk PCa since September 2019 (ACTRN:12619001669189). Unifocal ISUP GG1 (≥10 mm in ≥1 core), GG2 (any length) or GG3 (longest core<10 mm) were included. Follow‐up entailed serial PSA measurements, and surveillance mpMRI and repeat transperineal prostate biopsy at 18–24 months post‐treatment. Pathological control was achieved on repeat biopsy if there was no cancer or ISUP GG1 in <10 mm of core or GG2–3 grade cancer with radiation treatment effect. Progression was defined as no pathological changes from baseline or tumour upgrading.

**Results:**

Of 120 men enrolled, 55 (45.8%) have completed repeat histopathological assessments with a median (IQR) follow‐up of 38 (33–45) months. Pathological control was reported in 42 (76.4%) patients, including 25 negative biopsies, 12 clinically insignificant disease, and five in‐field ISUP GG2–3 with radiation treatment effect. Pathological progression was observed in 13 patients (23.6%), with concurrent clinically significant in‐ and out‐of‐field progression in three cases (5.5%) and isolated clinically significant out‐of‐field progression in 10 cases (18.2%). Five (9.1%) patients underwent salvage treatment, including three robotic‐assisted radical prostatectomies, one contralateral lobe LDR brachytherapy and one external beam radiation therapy. The salvage‐free survival at 1, 2, 3 and 4 years were 98.2%, 96.4%, 94.2% and 87.0%, respectively. Mean PSA velocity >0.55 ng/mL/year was a strong predictor of pathological progression (OR 23.54, 95% CI 4.28–129.35, *p* = 0.001), with a sensitivity of 76.9% and specificity of 90.5%.

**Conclusion:**

With a median follow‐up of 38 months, these early results suggest that focal LDR brachytherapy for low‐intermediate risk, single‐lesion, imaging‐visible PCa demonstrates satisfactory oncological control. However, further follow‐up is needed to assess long‐term oncological outcomes.

## 1 INTRODUCTION

Prostate cancer is the most common cancer affecting men in economically developed countries.^1^ Approximately 70% of men diagnosed with prostate cancer have organ‐confined disease, making them candidates for curative‐intent treatment through surgery or radiotherapy.^2^ However, these whole‐gland treatments can lead to significant morbidity, including urinary, bowel, and erectile toxicity.^3^ The 15‐year follow‐up results of the landmark ProtecT trial revealed that radical therapies did not provide any advantage in terms of prostate cancer‐specific and overall survival compared with expectant management.^4^ This underscores the importance of balancing treatment efficacy with the potential side effects of radical interventions, particularly for patients with low‐ to intermediate‐risk prostate cancer. Many of these patients may be placed on active surveillance to avoid or delay this morbidity; however, this has its limitations. Active surveillance may not be appropriate in some, as it may be associated with missing the window for treatment with curative intent in up to a quarter of the men whose disease eventually progresses.^5^ Additionally, it may cause psychological distress because of the perception of living with ‘untreated’ cancer.

Given the frequently multifocal nature of prostate cancer, whole‐gland treatment has traditionally been the standard of care.^6^ Clinicians have therefore focussed on improving existing whole‐gland radical therapies using minimally invasive methods, nerve‐sparing techniques and modern sculpted dosimetry. Yet, these efforts have yielded limited success in mitigating treatment‐related toxicity. The issue is that radical therapies carry a significant risk of damaging surrounding structures, including neurovascular bundles, bladder neck, prostatic urethra, external sphincter and rectum.^7^ Focal therapy is a proposed method to prevent such ‘collateral damage’. It is a hybrid approach that involves ablative treatment of the index lesion combined with ongoing surveillance of the untreated gland. Focal therapy offers a middle ground between active surveillance and whole‐gland radical therapies.

One concern with focal therapy is whether its oncological outcomes are non‐inferior to those of whole‐gland treatments. Reflecting this uncertainty, the 2024 European Association of Urology (EAU) guidelines recommend offering focal therapy only as part of a clinical trial or a well‐designed prospective registry.^8^ Nonetheless, many patients are willing to accept a potentially lower chance of cure in favour of better preservation of urinary and sexual function.^9^ This underscores the importance of shared decision‐making, where treatment choices are guided by individual preferences, values and tolerance for risk. Various ablative modalities are available for focal therapy, yet robust comparative data are lacking. We chose to study brachytherapy as an energy source for focal therapy given its long‐term status as a whole‐gland standard of care option for localised intermediate‐risk disease. Mohamad et al. recently published the largest systematic review and meta‐analysis on focal brachytherapy for localised prostate cancer.^10^ They found 10 studies over the past two decades with a total of 314 patients. They reported that the available evidence is highly limited because of heterogeneous definitions of failure, short follow‐ups and the small number of studies, which were mostly retrospective in nature with small sample sizes (range 15–51 patients). The current registry‐based study intends to provide further evidence on the efficacy of focal brachytherapy. We aimed to investigate the oncological control, pathological progression and its predictors following focal low‐dose‐rate (LDR) brachytherapy for low‐intermediate risk prostate cancer.

## 2 PATIENTS AND METHODS

### 2.1 Study design

LIBERATE is an ongoing, prospective, single‐arm, multi‐centre, IDEAL stage 2b, investigator‐led clinical registry of patients who have undergone focal LDR brachytherapy for low‐ to intermediate‐risk prostate cancer, as defined by the D'Amico Risk Classification, since September 2019 (ACTRN:12619001669189). The registry is sponsored by the Icon Cancer Foundation for infrastructural costs, such as study personnel for the collection of patient‐reported outcome measures (PROMs), multidisciplinary meetings, insurance and indemnity. It received ethics approval from the Bellberry Human Research Ethics Committee (Ref 2019‐09‐807). This study complies with the Strengthening the Reporting of Observational Studies in Epidemiology (STROBE) guidelines for reporting observational studies.^11^

### 2.2 Patient selection

Men were deemed eligible for focal LDR brachytherapy as part of LIBERATE registry if they met the following criteria including: an age of 40–85 years, life expectancy > 10 years, prostate‐specific antigen (PSA) < 15 ng/mL, clinical stage T1c or T2a, Prostate Imaging‐Reporting and Data System (PIRADS) score of 3–5 or suspicious prostate lesion on prostate‐specific membrane antigen positron emission tomography (PSMA‐PET), imaging‐concordant ISUP GG1 (≥10 mm in ≥1 core) or ISUP GG2 (any length) or ISUP GG3 (longest core <10 mm) prostate adenocarcinoma, and template biopsies of the remaining gland showing no cancer or clinically insignificant prostate cancer (<10 mm ISUP GG1), and ability to adhere to follow up protocol. Patients were excluded if they had suspicious or proven extra‐capsular extension, alternative histology (significant sarcomatoid, spindle cell, or neuroendocrine components; ductal adenocarcinoma or intraductal carcinoma of the prostate), other malignancy except for non‐melanoma skin cancer, anatomical abnormality or medical condition precluding brachytherapy or follow‐up imaging, and significant untreated lower urinary tract symptoms.

### 2.3 Disease localisation

The disease localisation was performed through multi‐parametric magnetic resonance imaging (mpMRI) and targeted as well as systematic transperineal biopsies. PSMA PET was utilised as an alternative for baseline and follow‐up imaging in those with contraindications to mpMRI, such as bilateral hip replacement, claustrophobia or incompatible pacemakers. PSMA PET may have also been performed in addition to mpMRI at the discretion of the referring urologist. mpMRI images were obtained with standard sequences, including T1‐, T2‐, and diffusion‐weighted imaging, and they were reported under PIRADS v.2 and v.2.1 conditions. Transperineal biopsies were conducted under general anaesthesia utilising a conventional 5 mm brachytherapy template grid and transrectal ultrasound (TRUS) probe. Targeting was achieved exclusively through cognitive fusion, and systematic grid biopsies were required to obtain at least 18 cores.

### 2.4 Technique

We have previously described our technique in detail.^12^ All patients underwent focal LDR brachytherapy by a single senior radiation oncologist using the three‐phase implant technique. This entailed pre‐planning volumetric assessment, seed implantation and post‐implantation dosimetric analysis. The TRUS‐guided volumetric assessment, performed around 2 weeks before treatment, assisted with seed planning and enabled the identification of any pubic arch interference. Software fusion of pre‐planning ultrasound and mpMRI was conducted using VariSeed (Varian Medical Systems, CA) by the radiation oncologist. This was subsequently verified by a senior radiation therapist or radiation oncology medical physicist. The focal gross tumour volume (F‐GTV) was the radiological extent of the index lesion. The focal planning target volume (F‐PTV) was defined as a 7‐mm isotropic expansion of the F‐GTV, providing a safety margin to account for potential underestimation of tumour extent on mpMRI.

The seed implantation was performed under general anaesthesia in an extended lithotomy position with real‐time intra‐operative dosimetric analysis within the VariSeed suite. Linked Iodine‐125 Amersham brachytherapy seeds were inserted under TRUS guidance. Additional ‘Zulu’ (free) seeds were inserted to ensure delivery of the prescribed dose of 145 Gy to the F‐PTV. A flexible cystoscopy was performed by a urologist at the end of the procedure to rule out the presence of any seeds within the urethra or bladder. Rectal spacer was injected as needed to minimise rectal toxicity. Routine urethral catheterisation was not performed; patients were observed postoperatively and discharged on the same day after successful voiding, with catheterisation reserved only for those who developed urinary retention. A non‐contrast pelvic computed tomography (CT) and mpMRI were obtained 4 weeks following seed insertion as part of post‐implantation dosimetric analysis.

### 2.5 Follow‐up

Follow‐up was conducted 6 weeks post‐treatment, every 3 months for the first 2 years, and then every 6 months up to 5 years after treatment. Reviews included a clinical examination, serial PSA measurement and toxicity assessments. Functional outcomes were evaluated via patient‐reported outcome measures using validated questionnaires (reported separately in a companion manuscript). Oncological outcomes were assessed with surveillance mpMRI and repeat transperineal prostate biopsy, with systematic and targeted biopsies, at 18–24 months post‐treatment. Pathological assessment was performed independently by pathologists at each participating site. In cases of diagnostic uncertainty, specimens were referred for secondary review by an experienced genitourinary pathologist with specific expertise in interpreting prostate biopsies following radiation therapy. Control was defined on repeat biopsy if there was no cancer or ISUP GG1 in <10 mm of core or GG2–3 cancer with radiation treatment effect. Progression occurred if there were no pathological changes from baseline or tumour upgrading occurred compared to baseline. Radiation treatment effect was defined histologically by the presence of post‐radiation changes such as glandular atrophy, cytoplasmic vacuolisation, nuclear pyknosis, smudged chromatin, stromal fibrosis and loss of prominent nucleoli.^13^ A Gleason score was assigned when residual viable tumour glands could be confidently assessed; otherwise, only treatment effect was reported. All patients with positive biopsies were discussed in a multidisciplinary meeting. The LIBERATE registry data were prospectively collected and quality‐controlled by dedicated research coordinators at the Icon Cancer Centre. For this study, the mean PSA velocity was calculated using values from consecutive 3‐ to 6‐month intervals, beginning at the 12‐month follow‐up to allow for PSA stabilisation after brachytherapy seed implantation, and continuing thereafter.

### 2.6 Statistical analysis

Normality was assessed with visual inspection of data distributions. Continuous variables were summarised as means with standard deviation (SD) or medians with interquartile range (IQR) based on their distribution, and categorical variables were presented as counts with percentages. Comparisons between patients with pathological progression and those with pathological control were conducted using the Student's *t*‐test or Mann–Whitney *U* test for continuous variables, and Pearson's Chi‐square or Fisher's exact test for categorical variables as appropriate. Multivariable logistic regression analysis was employed to identify predictors of pathological progression. Kaplan–Meier analyses were used to evaluate freedom from pathological progression and salvage treatment, with the log‐rank test applied to compare survival outcomes stratified by Gleason score and mean PSA velocity. Receiver operating characteristic (ROC) analysis was performed to determine the optimal mean PSA velocity cut‐off for predicting pathological progression, with the cut‐off point selected based on the highest Youden's Index to maximise sensitivity and specificity. All statistical analyses and their interpretation were independently reviewed by a statistician from Monash University. Statistical analyses were conducted using Stata software version 18.0 (StataCorp, TX), with two‐sided *p*‐values <0.05 considered statistically significant.

## 3 RESULTS

### 3.1 Baseline characteristics

Baseline characteristics are listed in Table 1. Of the 120 men enrolled in the LIBERATE Registry, 78 were eligible for repeat biopsy, of whom 55 completed repeat histopathological assessment. The remaining 23 either deferred or declined biopsy or were awaiting their scheduled procedure at the time of data cut‐off. Some patients declined re‐biopsy owing to marked PSA reduction and negative post‐treatment MRI, suggesting effective disease control. The median (IQR) follow‐up duration was 38 (33–45) months. This cohort had a mean ± SD age of 71.1 ± 7.2 years and a mean ± SD baseline PSA of 5.7 ± 2.2 ng/mL. Most patients had T1c clinical stage (89.1%) and a PIRADS 4 or 5 lesion on mpMRI (90.9%). On baseline transperineal biopsy, one (1.8%) patient had ISUP GG1, 47 (85.5%) ISUP GG2, and seven (12.7%) ISUP GG3 disease.

**TABLE 1:** Baseline characteristics.

Variable	Value
Number of patients, *n*	55
Follow up (months), median (IQR)	38 (33–45)
Baseline PSA (ng/mL), mean ± SD	5.7 ± 2.2
Baseline PSA free/total (%), mean ± SD	12.2 ± 7.0
Baseline PSA density (ng/mL^2^), median (IQR)	0.15 (0.11–0.20)
Clinical stage, *n* (%)	
T1c	49 (89.1)
T2a	6 (10.9)
Age (years), mean ± SD	71.1 ± 7.2
BMI (kg/m^2^), median (IQR)	27.0 (25.8–29.4)
PIRADS score, *n* (%)	
2 and suspicious lesion on PSMA PET	1 (1.8)
3	3 (5.5)
4	40 (72.7)
5	10 (18.2)
No MRI but suspicious PSMA PET	1 (1.8)
Lesion location, *n* (%)*	
Anterior	27 (29.0)
Mid	30 (32.3)
Posterior	36 (38.7)
Prostate volume (cc), median (IQR)	39.0 (29.0–50.0)
Suspicious lesion on PSMA PET, *n* (%)	11 (20.0)
SUV max, median (IQR)	5.0 (3.1–10.3)
Baseline transperineal biopsy, *n* (%)	
ISUP GG1	1 (1.8)
ISUP GG2	47 (85.5)
ISUP GG3	7 (12.7)
Baseline transperineal biopsy	
Total number of cores taken, median (IQR)	29 (25–34)
Target number of cores taken, median (IQR)	6 (5–7)
Template number of cores taken, median (IQR)	24 (19–29)
Longest length cancer (mm), mean ± SD	7.3 ± 3.5

Abbreviations: *n*, number; IQR, interquartile range; SD, standard deviation; PSA, prostate‐specific antigen; BMI, body mass index; PIRADS, prostate imaging‐reporting and data system; PSMA PET, prostate‐specific membrane antigen positron emission tomography; MRI, magnetic resonance imaging; SUV max, maximum standardised uptake value; ISUP GG, International Society of Urological Pathology grade group.

* Several lesions crossed into multiple zones.

### 3.2 Dosimetry

The median (IQR) prostate volume was 41.5 cc (11–103). Most men (87.3%) had rectal spacer inserted. The median (IQR) number of seeds used was 27 (23–33), with a mean ± SD total implanted activity of 10.7 ± 3.5 mCi. The mean focal‐planning target volume (F‐PTV) was 8.1 cc (range 2.0–19.0 cc), representing 19.4% (range 4.6–66.8%) of the total prostate volume. The mean post‐implantation V100% and D90% for the F‐GTV were 99.8% (range 92.7–100%) and 277.1 Gy (range 150.5–434.2 Gy), respectively (Table 2).

**TABLE 2:** Dosimetry outcomes.

Variable	Value
Rectal spacer insertion, *n* (%)	48 (87.3)
Number of seeds, median (IQR)	27 (23–33)
Total implanted activity (mCi), mean ± SD	10.7 ± 3.5
Geometry, mean [range]	
Prostate volume (cc)	41.5 [11–103]
F‐GTV (cc)	2.2 [0.1–6.4]
F‐PTV (cc)	8.1 [2.0–19.0]
F‐PTV (% of prostate volume)	19.4 [4.6–66.8]
F‐GTV, mean [range]	
V100% (%)	99.8 [92.7–100]
V150% (%)	96.0 [52.0–100]
D90% (Gy)	277.1 [150.5–434.2]
Prostate, mean [range]	
V100% (%)	24.6 [8.1–71.5]
Urethra, mean [range]	
Max (Gy)	183.3 [39.9–462.8]
V200% (cc)	0.01 [0.00–0.14]
Rectum, mean [range]	
Max (Gy)	49.6 [19.4–142.5]
V100% (cc)	0.01 [0.00–0.50]

Abbreviations: *n*, number; IQR, interquartile range; mCi, millicurie; SD, standard deviation; F‐GTV, focal gross tumour volume; F‐PTV, focal planning target volume; V100%, volume receiving 100% of the prescribed dose; V150%, volume receiving 150% of the prescribed dose; Gy, Grey; D90%, dose to 90% of the structure volume; V200%, volume receiving 200% of the prescribed dose.

### 3.3 Follow‐up patient outcomes

**FIGURE 1:**
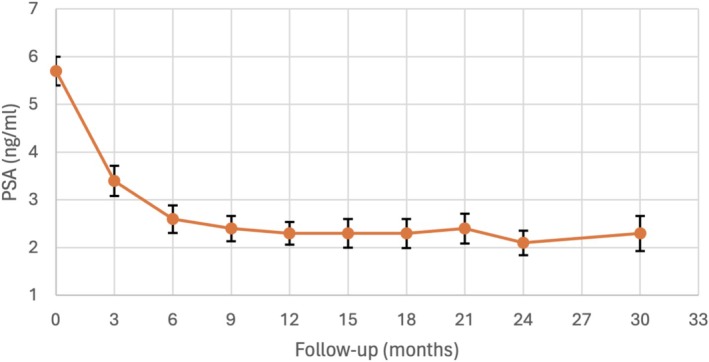
Graph depicting the kinetics of mean PSA following focal LDR brachytherapy and its standard error. PSA data following salvage treatments were excluded.

Table 3 describes follow‐up patient outcomes. Most men had no concerning lesions on repeat MRI (87.3% PIRADS 2). The median (IQR) time from seed insertion to repeat biopsy was 19 (18–20) months. The median (IQR) PSA nadir was 1.2 (0.6–2.2) ng/mL, and the mean ± SD PSA 18 months following seed insertion was 2.3 ± 2.0 ng/mL. Figure 1 depicts mean serial PSA measurements.

**TABLE 3:** Follow‐up imaging, biochemical and histopathological outcomes.

Variable	Value
Follow‐up imaging, *n* (%)	
Repeat MRI	
PIRADS 2	48 (87.3)
PIRADS 3	2 (3.6)
PIRADS 4	2 (3.6)
PIRADS 5	0 (0)
Repeat PSMA PET	
MRI contraindicated, no lesions on repeat PSMA PET	1 (1.8)
PIRADS4 and suspicious lesion on repeat PSMA PET	1 (1.8)
No repeat imaging	1 (1.8)
Time from implant to repeat biopsy (months), median (IQR)	19 (18–20)
Time from initial biopsy to re‐biopsy (months), median (IQR)	22 (21–24)
PSA 18 months post‐implant (ng/mL), mean ± SD	2.3 ± 2.0
PSA nadir (ng/mL), median (IQR)	1.2 (0.6–2.2)
Follow‐up histopathology, *n* (%)	
Repeat transperineal biopsy	
Negative biopsy	25 (45.5)
ISUP GG1	12 (21.8)
ISUP GG2	12 (21.8)
ISUP GG3	3 (5.5)
Radical prostatectomy specimen	
ISUP GG3	1 (1.8)
Ungraded acinar adenocarcinoma with significant radiotherapy effect	1 (1.8)
Ungraded acinar adenocarcinoma with significant radiotherapy effect (features of ISUP GG5)	1 (1.8)
Clinical significance, *n* (%)	
Pathological progression	
Concurrent CS in‐ and out‐of‐field progression	3 (5.5)
Isolated CS in‐field progression	0 (0)
Isolated CS out‐of‐field progression	10 (18.2)
Pathological control	
Negative biopsy	25 (45.5)
In‐field GG2–3 with treatment effect	5 (9.1)
Clinically insignificant disease (ISUP GG1 < 10 mm)	12 (21.8)

Abbreviations: *n*, number; MRI, magnetic resonance imaging; PIRADS, prostate imaging‐reporting and data system; PSMA PET, prostate‐specific membrane antigen positron emission tomography; IQR, interquartile range; PSA, prostate‐specific antigen; ISUP GG, International Society of Urological Pathology grade group; CS, clinically significant.

**FIGURE 2:**
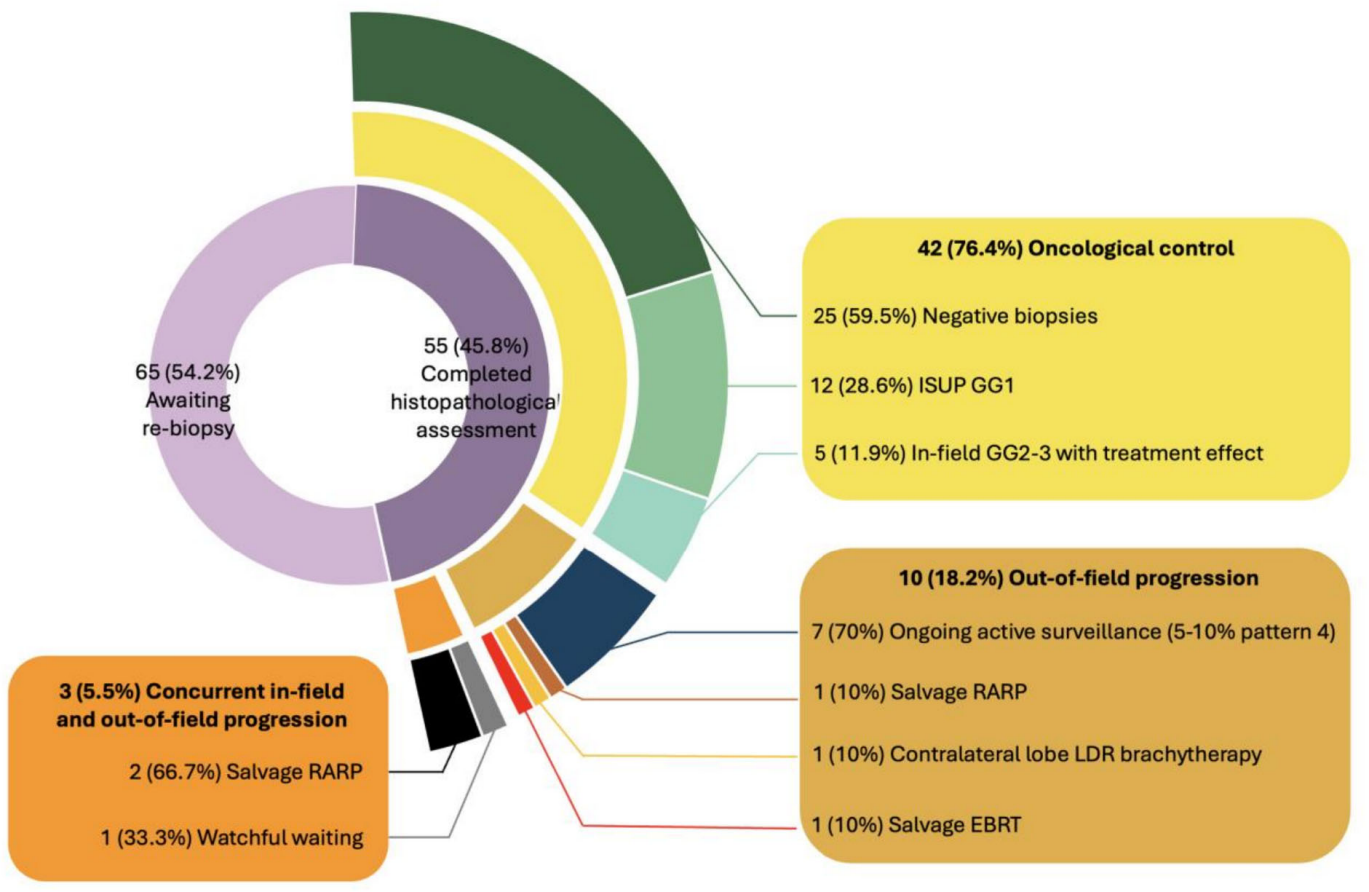
Pie chart depicting classification based on histopathological assessment and corresponding treatment provided. RARP, robotic‐assisted radical prostatectomy; ISUP GG, International Society of Urological Pathology grade group; LDR, low‐dose‐rate; EBRT, external beam radiation therapy.

Pathological control was achieved in 42 patients (76.4%), all of whom were managed with ongoing surveillance, including 25 (45.5%) negative biopsies, five (9.1%) in‐field ISUP GG 2–3 with radiation treatment effect and 12 (21.8%) clinically insignificant disease (ISUP GG1 < 10 mm) (Figure 2). Pathological progression was observed in 13 patients (23.6%), with concurrent clinically significant in‐ and out‐of‐field progression in three cases (5.5%) and isolated clinically significant out‐of‐field progression in 10 cases (18.2%). There were no instances of isolated clinically significant in‐field progression. Additionally, no patients had persistent in‐field lesions without treatment effect.

Among the 13 patients with pathological progression on repeat biopsy, seven were managed with ongoing active surveillance (all with 5–10% Gleason pattern 4), one was transitioned to watchful waiting, two underwent salvage robotic‐assisted radical prostatectomy (RARP), one had contralateral lobe LDR brachytherapy and one proceeded to external beam radiation therapy. For those remaining on active surveillance, the median percentage of Gleason pattern 4 decreased from 20% at baseline to 5% post‐treatment, and the median greatest length of positive cores decreased from 5 to 1.5 mm. One patient proceeded to salvage RARP without repeat biopsy because of a rising PSA and strong suspicion of a new index lesion on imaging. He was found to have concurrent in‐ and out‐of‐field progression. During the follow‐up period, no patients developed metastasis or needed systemic treatment.

### 3.4 Progression‐free and salvage‐free survival

**FIGURE 3:**
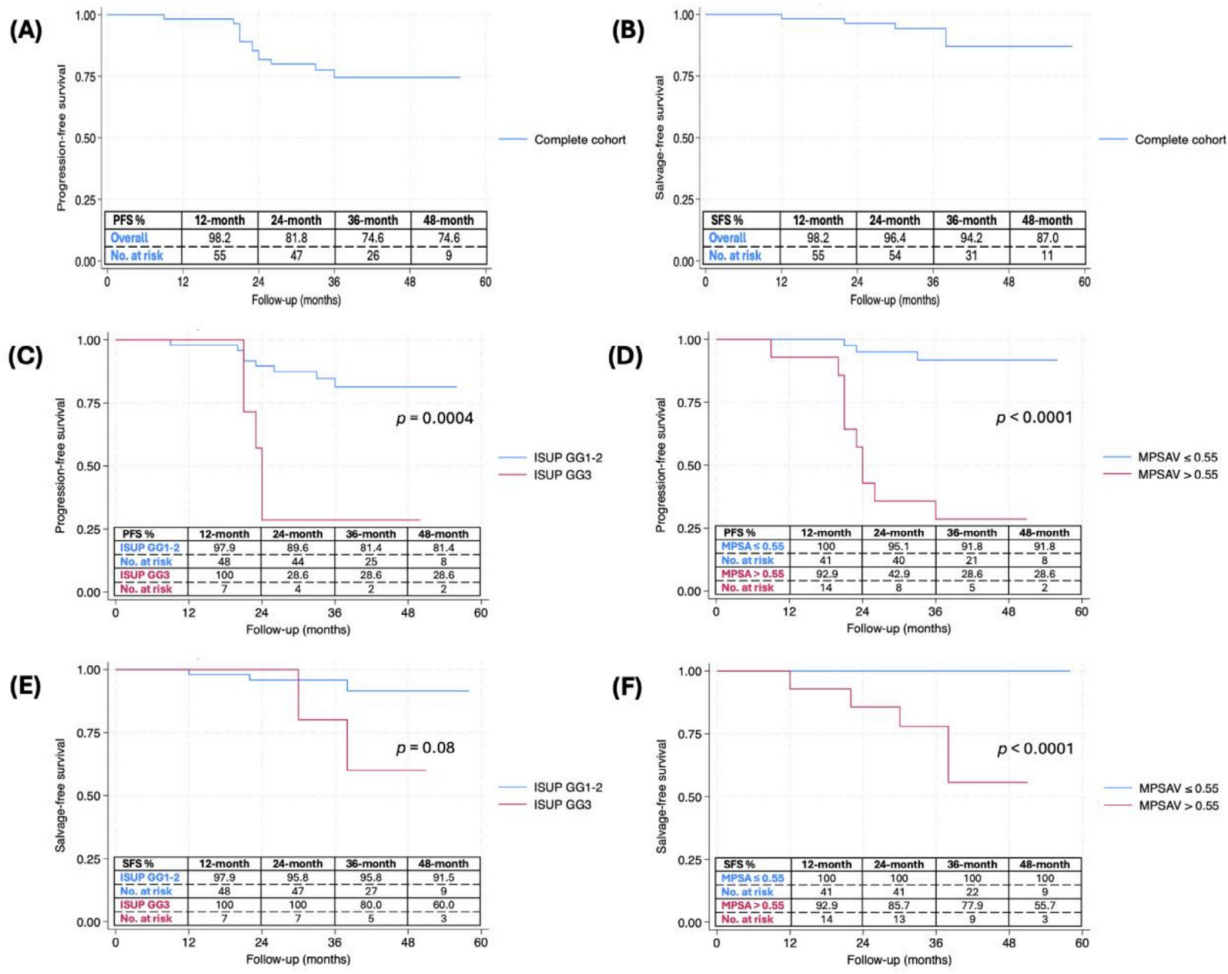
Kaplan–Meier curves showing (A) progression‐free survival for the entire cohort, (B) salvage‐free survival for the entire cohort, (C) progression‐free survival by Gleason score, (D) progression‐free survival by mean PSA velocity, (E) salvage‐free survival by Gleason score and (F) salvage‐free survival by mean PSA velocity. PFS, progression‐free survival; SFS, salvage‐free survival; No., number; ISUP GG, ISUP grade group; MPSAV, mean PSA velocity.

The Kaplan–Meier survival curves for progression‐free and salvage‐free survival are depicted in Figure 3. The salvage‐free survival rates at 1, 2, 3, and 4 years were 98.2% (95%CI 87.8–99.7%), 96.4% (95%CI 86.2–99.1%), 94.2% (95%CI 83.1–98.1%) and 87.0% (95%CI 70.2–94.7%), respectively. The progression‐free survival rates at 1, 2, 3 and 4 years were 98.2% (95%CI 87.8–99.7%), 81.8% (95%CI 68.8–89.8%), 74.6% (95%CI 59.8–84.6%) and 74.6% (95%CI 59.8–84.6%), respectively. Patients with higher Gleason scores (ISUP GG3 compared to GG1–2) exhibited significantly worse progression‐free survival (*p* = 0.0004) and poorer salvage‐free survival, although the latter did not reach statistical significance (*p* = 0.08).

### 3.5 Predictors of pathological progression

Univariate analysis demonstrated that more aggressive histology and various markers of unfavourable PSA dynamics were significantly associated with an increased likelihood of pathological progression (Table 4). A higher proportion of patients with pathological progression had ISUP GG3 on baseline biopsy than those with pathological control (38.5% versus 4.8%, *p* = 0.001). Patients with pathological progression exhibited a higher mean PSA velocity than those with pathological control (+1.20 versus −0.06, *p* = 0.007).

**TABLE 4:** Univariate and multivariable analysis to predict pathological progression.

	Pathological progression (*n* = 13)	Pathological control (*n* = 42)	*p*‐value	Multivariate analysis	
				Odds ratio (95% CI)	*p* value
Higher Gleason score (ISUP GG3 vs GG1–2), *n* (%)	5 (38.5)	2 (4.8)	0.001^a^	5.31 (0.53–53.50)	0.2
Age (years), mean ± SD	71.6 ± 9.6	71.0 ± 6.4	0.8		
Prostate volume (cc), median (IQR)	31.0 (28.0–61.0)	40.0 (30.0–47.0)	0.9		
BMI (kg/m^2^), median (IQR)	26.9 (25.9–28.6)	27.0 (25.3–29.4)	0.9		
Positive follow‐up MRI (PIRADS 4 vs 2–3), *n* (%)	2 (15.4)	1 (2.4)	0.1		
Higher clinical stage, (T2a vs T1c), *n* (%)	1 (7.7)	5 (11.9)	0.7		
Baseline PSA (ng/mL), mean ± SD	6.2 ± 1.9	5.6 ± 2.3	0.4		
Baseline PSA density (ng/ml/cc), median (IQR)	0.18 (0.11–0.25)	0.14 (0.11–0.19)	0.5		
Baseline PSA free/total ratio (%), mean ± SD	13.1 ± 6.2	11.9 ± 7.4	0.7		
PSA 21‐months post seed insertion, (ng/mL^2^), mean ± SD	3.2 ± 2.6	2.2 ± 1.8	0.1		
PSA nadir (ng/mL), median (IQR)	1.1 (0.7–2.2)	1.2 (0.5–2.4)	0.9		
Proportion of PSA reduction from baseline to nadir (%), median (IQR)	−82.4 (−89.7 –−69.6)	−75.3 (−87.2 –−63.3)	0.5		
Mean PSA velocity (ng/mL/year), mean ± SD	+ 1.20 ± 2.06	‐ 0.06 ± 1.16	0.007^b^		
Mean PSA velocity > 0.55 ng/mL/year, *n* (%)	10 (76.9)	4 (9.5)	<0.0001^a^ ^,^ ^b^	23.54 (4.28–129.35)	0.001
Two consecutive PSA increases ≥0.5 ng/mL, *n* (%)	6 (46.2)	3 (7.1)	0.001^b^		

Abbreviations: 95% CI; 95% confidence interval, *n*, number; ISUP GG, International Society of Urological Pathology grade group; SD, standard deviation; IQR, interquartile range; BMI, body mass index; MRI, magnetic resonance imaging; PIRADS, prostate imaging‐reporting and data system; PSA, prostate‐specific antigen.

^a^ Statistically significant factors included in multivariate analysis.

^b^ Given the high correlation between these three PSA dynamic markers, to avoid collinearity, only mean PSA velocity > 0.55 ng/mL/year, which was the strongest predictor of pathological progression, was included in the multivariate analysis.

**FIGURE 4:**
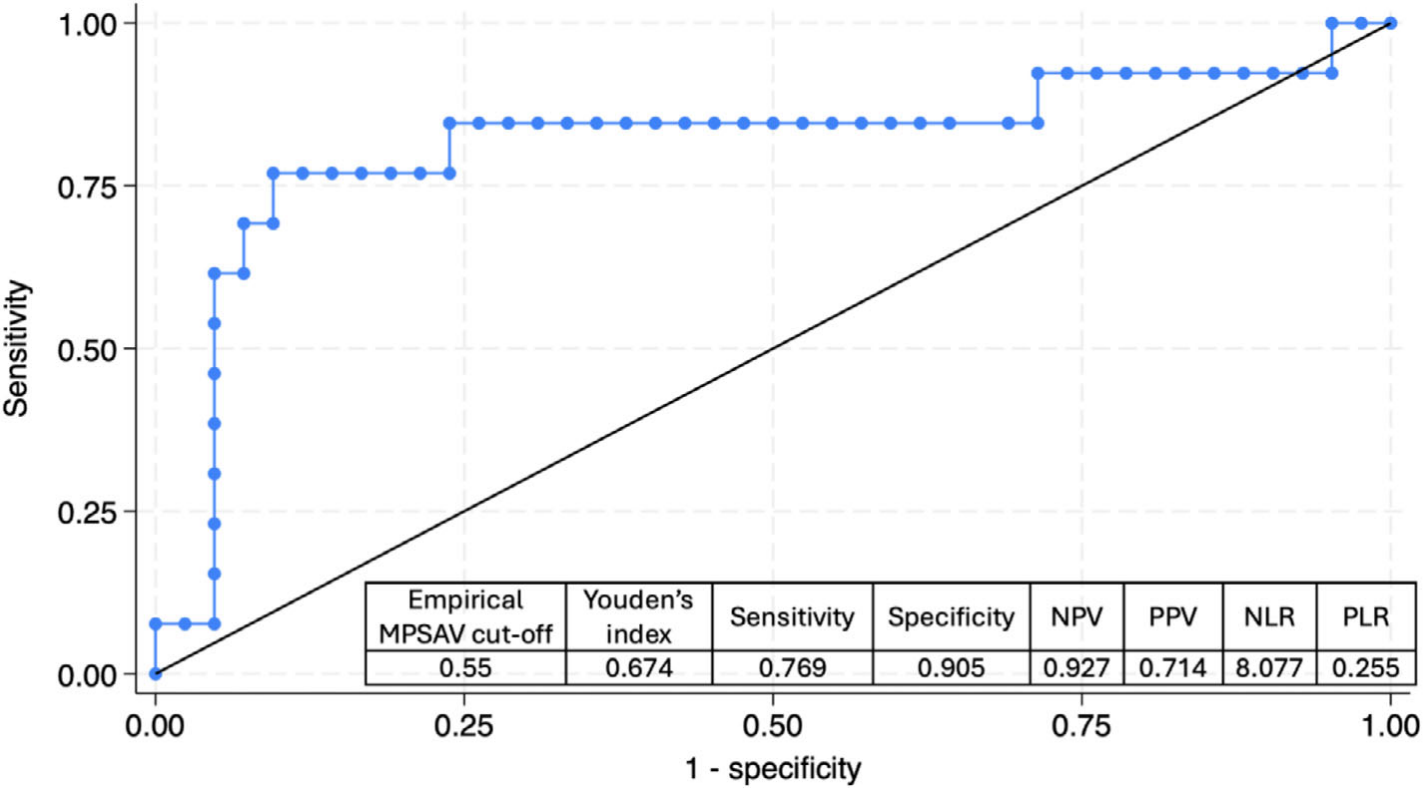
Receiver operating characteristic (ROC) curve for different mean PSA velocity cut‐off values to predict pathological progression. The area under the ROC curve was 0.815 (95% CI 0.644–0.986). The 0.55 ng/mL/year cut‐off for mean PSA velocity yielded the highest Youden's index. MPSAV, mean PSA velocity; NPV, negative predictive value; PPV, positive predictive value; NLR, negative likelihood ratio; PLR, positive likelihood ratio.

The area under the ROC curve for different mean PSA velocity cut‐off values to predict pathological progression was 0.815 (95% CI 0.644–0.986), demonstrating excellent discriminative power (Figure 4). A mean PSA velocity cut‐off value of 0.55 ng/mL/year yielded the highest Youden's index (0.674) with a sensitivity of 76.9% and a specificity of 90.5% for predicting pathological progression. Although three PSA dynamic markers were significant predictors of pathological progression in the univariate analysis (Table 4), their high correlation necessitated including only one of them in the multivariate model to avoid collinearity. Therefore, only the strongest predictor (i.e. mean PSA velocity >0.55 ng/mL/year) was included.

Multivariate analysis found that after adjusting for the Gleason score, a mean PSA velocity of >0.55 ng/mL/year remained an independent predictor of pathological progression (OR 23.54, 95%CI 4.28–129.35, *p* = 0.001). Kaplan–Meier analysis also showed that progression‐free and salvage‐free survival were significantly different when stratified based on a mean PSA velocity of >0.55 ng/mL/year (*p* < 0.0001). No patient with a mean PSA velocity of ≤0.55 ng/mL/year needed salvage therapy over the follow‐up period (Figure 3).

## 4 DISCUSSION

With a median follow‐up of 38 months, we observed a pathological control rate of 76.4%, comprising 45.5% negative biopsies, 21.8% clinically insignificant disease (ISUP GG1 < 10 mm), and 9.1% in‐field ISUP GG 2–3 with radiation treatment effect. Pathological progression occurred in 23.6% of patients, primarily as isolated out‐of‐field progression (18.2%), with concurrent in‐ and out‐of‐field progression in 5.5%. This predominance of out‐of‐field progression highlights the critical role of serial imaging and systematic biopsy in identifying concurrent or de novo multifocal significant disease.

The observed rates of pathological progression (23.6%) and salvage whole‐gland treatment (9.1%) in our study align well with prior studies on focal LDR brachytherapy. Matsuoka et al. evaluated outcomes in 51 patients with low‐ to intermediate‐risk prostate cancer who underwent focal LDR brachytherapy, reporting clinically significant recurrence in 13 patients (25.5%), salvage focal therapy in 10 (19.6%), and salvage whole‐gland treatment in four (7.8%) over a median follow‐up of 5.7 years.^14^ Similarly, Ta et al., in a study of 39 men with low‐ to intermediate‐risk prostate cancer treated with focal LDR brachytherapy, documented out‐of‐field progression in seven patients (21%) and salvage whole‐gland therapy in four (11.8%) after a mean follow‐up of 65 months.^15^ Saito et al. investigated hemi‐gland LDR brachytherapy in 24 patients with intermediate‐risk disease, reporting significant prostate cancer recurrence in the treated lobe in one patient (4%) and untreated lobes in four patients (17%).^16^ Among these, five patients (20.8%) underwent salvage treatment, including three cases of re‐focal brachytherapy, one radical prostatectomy and one androgen deprivation therapy. Our pathological progression rate is also consistent with the recurrence rates observed in other forms of focal therapy (15–30%) across different studies with large cohorts.^17^, ^18^, ^19^

Focal LDR brachytherapy offers several advantages over other focal therapy energy sources. Its effectiveness has been well‐established in the whole‐gland setting, a distinction not shared by other energy sources. While research continues to explore the efficacy of high‐intensity focussed ultrasound (HIFU) and cryoablation as whole‐gland treatments, the EAU guidelines still classify them as experimental at this time.^8^, ^20^, ^21^, ^22^ In contrast, numerous studies with long‐term follow‐up have demonstrated favourable oncological and functional outcomes for patients treated with whole‐gland LDR brachytherapy for prostate cancer.^23^, ^24^, ^25^ Furthermore, focal LDR brachytherapy is the only focal therapy modality eligible for public funding in Australia (item number 37220), offering a significant financial advantage by reducing the economic burden on patients.^26^

Interpreting prostate mpMRI following focal therapy presents distinct challenges because of treatment‐related tissue changes that may mimic or obscure residual disease. Conventional scoring systems such as PI‐RADS, originally developed for treatment‐naive glands, are suboptimal in this context, prompting the development of post‐treatment frameworks such as PI‐FAB (Prostate Imaging after Focal Ablation).^27^ While PI‐FAB provides a structured approach for interpreting mpMRI after ablative modalities, mainly HIFU, its performance in the setting of radiotherapy‐based focal therapies remains insufficiently validated. Seed‐related artefacts, non‐thermal tissue responses and heterogeneous dose distribution may alter mpMRI characteristics differently compared with ablative modalities. As our study predated the release of PI‐FAB and given these limitations, we utilised PI‐RADS as a familiar, pragmatic and reproducible framework for lesion assessment within the context of focal LDR brachytherapy.

We defined pathological control to include patients with ISUP GG2–3 prostate cancer exhibiting significant radiation‐induced treatment effects. This is because of the potential delay in complete histological resolution of the original Gleason pattern secondary to radiation‐induced post‐mitotic cell death, which may exceed the protocolled 18‐month interval between treatment and repeat biopsies in our study.^28^, ^29^ This approach is supported by existing literature, which shows that patients with persistent Gleason patterns with significant radiation‐induced treatment changes achieve oncological outcomes equivalent to those with negative biopsies (no evidence of carcinoma). Conversely, patients with prostate adenocarcinoma lacking typical radiation‐induced changes exhibit oncological behaviour similar to those with positive biopsies.^30^

Our study revealed that patients with ISUP GG3 had the highest rates of pathological progression and an increased likelihood of requiring salvage treatment following focal therapy. These findings are in keeping with the literature which suggests that GG3 prostate cancer demonstrates more aggressive biology and a higher risk of recurrence compared with GG2.^19^, ^31^ Nevertheless, focal therapy remains acceptable for carefully selected men who, after thorough counselling, prioritise functional preservation and are fully informed of the higher oncologic risks and the potential need for secondary treatment. Patient preference and individual risk tolerance play a central role in treatment decision‐making.^32^ Patients should be educated about the inherent oncological trade‐offs associated with focal therapy, which prioritises superior functional outcomes at the potential cost of increased surveillance demands. Active surveillance following focal therapy is integral to its success; therefore, patients who are unwilling or unable to adhere to a rigorous surveillance protocol may be better suited to whole‐gland treatment options and should not routinely be offered focal therapy.

The single‐arm design precludes direct comparison with radical whole‐gland therapies; however, the present findings support the oncologic safety of focal LDR brachytherapy in appropriately selected men. No isolated in‐field progression was observed, and only 9.1% of patients required salvage treatment over 38 months. Contemporary series report that approximately 22–24% of men with intermediate‐risk prostate cancer treated with radical prostatectomy experience biochemical recurrence within 5 years, potentially necessitating salvage radiotherapy.^33^ Similarly, patients with intermediate‐risk prostate cancer treated with external beam radiotherapy demonstrate a comparable 20% rate of Phoenix‐defined biochemical failure within 5 years, most commonly managed with salvage androgen deprivation therapy.^34^ Compared with active surveillance, focal LDR brachytherapy aims to reduce the chance of disease progression while avoiding the global toxicity of radical treatment. For instance, in a randomised trial, focal photodynamic therapy significantly reduced the subsequent detection of higher‐grade cancer on repeat biopsy and lowered the likelihood of conversion to radical treatment compared with active surveillance (24% vs 53% at 4 years; hazard ratio 0.31).^35^ Focal therapy is best framed as a disease‐modifying strategy that balances intent for oncologic control with functional preservation, rather than a wholesale substitute for radical therapy or a default alternative to active surveillance. For patients who want to actively treat their cancer, but do not want risk the side effects of standard radical therapy, only focal therapy provides patients with a third alternative between the limiting binary standard options.

Serial PSA measurement remains a critical tool for identifying patients with residual disease following focal therapy; however, there is currently no consensus on a definition for biochemical recurrence in this context. The Phoenix criteria, defined as a rise in PSA of ≥2 ng/mL above the nadir, is commonly used metric but is less reliable post focal therapy.^36^ Unlike whole‐gland treatments, focal therapy targets specific sub‐regions of the prostate, leaving untreated benign prostatic tissue that can undergo hyperplasia and release increasing levels of PSA. As a result, the PSA level may not decrease as significantly or fluctuate, complicating its interpretation. In our study, a mean PSA velocity >0.55 ng/mL/year emerged as the strongest independent predictor of pathological progression, demonstrating moderate sensitivity (76.9%) and high specificity (90.5%). This aligns with findings by Nguyen et al., who suggested a PSA velocity >0.75 ng/mL/year as a potential marker of biochemical failure.^37^ Alternative metrics have also been proposed, including the historical American Society for Radiation Oncology (ASTRO) consensus criteria, defined as three consecutive rises in PSA levels following the nadir.^38^ Further research is required to externally validate the utility and accuracy of our proposed mean PSA velocity threshold. These efforts could provide a more reliable framework for detecting biochemical recurrence and guiding subsequent management.

To the best of our knowledge, this is the largest study to date on focal brachytherapy for prostate cancer. A key strength of this study is its rigorous follow‐up protocols, with all patients undergoing repeat histopathological assessments. Unlike many similar studies that primarily report biochemical recurrence, this study incorporates routine tissue‐based assessments, providing a more accurate evaluation of pathological progression. Studies that relied solely on serial PSA measurements and imaging, with biopsies performed only as clinically indicated, may have underestimated the true rate of progression by failing to detect MRI‐occult disease or progression without significant PSA elevation. Furthermore, this study benefits from prospectively collected data within a well‐designed registry setting, incorporating comprehensive oncological and functional assessments. This robust data collection framework significantly enhances the reliability and validity of the findings, distinguishing it from prior research in the field.

This study was limited by its non‐randomised, single‐arm study design and relatively small sample size because of stringent selection criteria. Although all patients underwent seed insertion by a single expert radiation oncology team, pre‐treatment and follow‐up biopsies were performed by referring community urologists who did not have access to fusion software and therefore relied exclusively on cognitive targeting. Hence, different biopsy techniques may have influenced our results; however, this enhances the generalisability of the findings to real‐world clinical practice. Furthermore, the follow‐up period of this study allows for only medium‐term outcome evaluations, and long‐term oncological outcomes remain to be determined.

In conclusion, with a median follow‐up of 38 months, these early results suggest that focal LDR brachytherapy for low‐intermediate risk, single‐lesion and imaging‐visible prostate cancer provides satisfactory oncological control while enabling the early detection of treatment failure and timely decision‐making for further interventions. The findings reinforce the importance of PSA dynamics, particularly mean PSA velocity >0.55 ng/mL/year, as a robust predictor of pathological progression, offering a valuable tool for post‐treatment monitoring. Larger multicentre randomised trials with extended follow‐up are needed to validate these findings and assess long‐term oncological outcomes.

## AUTHOR CONTRIBUTIONS

*Conceptualisation and study design*: Mohammadmehdi Adhami, Jeremy Cheng, Elliot Anderson, Lloyd Smyth, Nathan Lawrentschuk, Jeremy Grummet and Andrew See. *Data Acquisition*: Mohammadmehdi Adhami, Elliot Anderson, Lloyd Smyth, Cate Davey, Thang Nguyen, Jeremy Grummet and Andrew See. *Data analysis and interpretation*: Mohammadmehdi Adhami, Jeremy Grummet. *Manuscript Writing*: Mohammadmehdi Adhami and Jeremy Cheng. *Manuscript review*: Mohammadmehdi Adhami, Jeremy Cheng, Elliot Anderson, Lloyd Smyth, Cate Davey, Thang Nguyen, Richard O'Sullivan, Andrew Ryan, Nathan Lawrentschuk, Jeremy Grummet and Andrew See. All authors have reviewed the manuscript and approved the final version for publication.

## CONFLICT OF INTEREST STATEMENT

The authors declare no conflicts of interest.
